# Conserved eukaryotic factors XCT and COP1 work together to control circadian clock function and reproductive timing in plants

**DOI:** 10.1038/s44323-025-00057-x

**Published:** 2026-01-09

**Authors:** Roderick W. Kumimoto, Carine M. Marshall, Hongtao Zhang, Stacey L. Harmer

**Affiliations:** Department of Plant Biology, University of California, Davis, CA USA

**Keywords:** Circadian rhythms in plants, Experimental organisms, Developmental biology

## Abstract

Circadian rhythms align biological processes with environmental cycles to enhance fitness. Eukaryotic circadian oscillators rely on transcriptional–translational feedback loops, although specific oscillator components vary among lineages. The plant oscillator consists of a uniquely complex network of transcriptional regulators. These clock genes regulate each other’s expression and influence key developmental processes such as the transition from vegetative to reproductive growth. Here, we investigate the function of XAP5 CIRCADIAN TIMEKEEPER (XCT), a protein conserved across eukaryotes and previously implicated in RNA processing and circadian regulation in *Arabidopsis thaliana*. We show that XCT genetically and physically interacts with CONSTITUTIVE PHOTOMORPHOGENIC 1 (COP1), an E3 ubiquitin ligase also conserved across eukaryotes. Both XCT and COP1 regulate the abundance and activity of the oscillator component EARLY FLOWERING3 (ELF3) to modulate circadian function and flowering time. These findings demonstrate how broadly conserved factors collaborate with plant-specific regulators to maintain circadian integrity and coordinate developmental timing.

## Introduction

Circadian rhythms, daily rhythms in physiology or behavior, are ubiquitous in nature and have been shown to promote fitness in bacteria, plants, and animals^1–4^. Although many circadian rhythms persist in constant environmental conditions, they most likely evolved due to their ability to phase diverse biological events to the most appropriate times of day^5^. Given the changing day lengths across the seasons, this requires the continual resetting of the circadian clock by environmental cues in a process termed entrainment. A key entrainment cue is light, which in plants is sensed by an impressive array of photoreceptors. Much is now known about how the downstream light signaling pathways affect circadian clock components and thus promote entrainment^6^. Importantly, although alterations in photoreceptors and downstream signaling factors can affect clock pace, light signaling components are not considered intrinsic constituents of the circadian machinery.

At the core of the plant circadian oscillator is an exceedingly complex network of transcriptional regulators that control each other’s expression. There are many positive and negative feedback loops and considerable genetic redundancy between the 20 or so key players^7^, but some important interactions are described here. The morning-phased transcription factors CIRCADIAN CLOCK ASSOCIATED 1 (CCA1) and LATE ELONGATED HYPOCOTYL (LHY) directly repress expression of clock genes, including the *PSEUDO-RESPONSE REGULATOR* (*PRR*) family (*PRR9,7,5,3* and *TIMING OF CAB EXPRESSION 1* (*TOC1*))^8,9^. The PRRs and TOC1 directly repress expression of *LUX ARRHYTHMO* (*LUX*), *EARLY FLOWERING 3* (*ELF3*), and *ELF4*, three genes that encode components of the evening complex (EC)^10,11^. The EC in turn represses expression of *PRR7*, *PRR9*, and *LUX*^12–14^. In addition to these repressors, additional essential clock genes such as LIGHT-REGULATED WD1 (LWD1), REVEILLE 8 (RVE8), and NIGHT LIGHT-INDUCIBLE AND CLOCK-REGULATED (LNK) proteins promote transcription of clock genes and are themselves transcriptionally regulated by each other and other clock components^15–17^. Post-translational regulation is also essential for proper clock function^7^, with the co-chaperone-type protein GIGANTEA (GI) and the photoreceptor/F-box protein ZEITLUPE (ZTL) playing key roles.

In addition to regulating each other’s expression, core clock proteins directly and indirectly control the expression of thousands of output genes that influence many aspects of growth and development^18^. One key output gene is *CONSTANS* (*CO*), a rhythmically expressed transcript that plays an important role in regulating the transition from vegetative to reproductive growth. In *Arabidopsis thaliana* and other long-day plants, the CO protein is stabilized when peak *CO* mRNA levels occur during daylight hours but is destabilized in short-day conditions. In long days, CO protein accumulates and promotes expression of *FLOWERING LOCUS T* (*FT*), which acts at the shoot apical meristem to induce flowering. As a key regulator of flowering, *CO* is subject to considerable regulation at the transcriptional, post-transcriptional, and post-translational levels^19^. Circadian clock components play many roles in this complex regulatory process; for example, GI promotes *CO* transcription while ELF3 promotes degradation of CO protein^20–23^.

Light signaling pathways directly regulate flowering time pathway components in addition to affecting flowering time via circadian clock entrainment. For example, the blue light photoreceptor CRYPTOCHROME 2 (CRY2) both promotes *CO* and *FT* transcription and inhibits degradation of CO protein^24–26^. CRY2 accomplishes the latter by inhibiting the activity of CONSTITUTIVE PHOTOMORPHOGENIC 1 (COP1). COP1 is a RING finger E3 ubiquitin ligase that ubiquitinates CO to promote its degradation by the 26S proteasome in the dark^25,27^. COP1 also promotes the degradation of GI in an ELF3-dependent manner, thus indirectly negatively regulating CO accumulation^23^. COP1 also affects circadian clock pace, presumably by modulation of light input pathways to the circadian system^28^. Although first identified for its role in the regulation of plant photomorphogenesis, COP1 orthologs in animals have since been recognized for their important roles in mammalian metabolism and tumorigenesis^29,30^.

Another protein well-conserved across eukaryotes that has been implicated in photomorphogenesis and circadian clock function in plants and tumorigenesis in animals is XAP5 CIRCADIAN TIMEKEEPER (XCT)^31–33^. *xct* mutants were first identified on the basis of their short circadian periods, but XCT and its orthologs were subsequently found to modulate RNA processing in animals and plants and gene expression in *Chlamydomonas reinhardtii* and fission yeast^34–38^. Although XCT orthologs do not possess any recognizable functional domains, they localize to the nucleus and exhibit a high degree of sequence conservation across eukaryotes^33,34,38,39^.

In previous work, we presented data suggesting that the RNA processing and circadian clock phenotypes observed in Arabidopsis plants mutant for *XCT* are separable^36^. Here, we further investigate the role of XCT in the plant circadian system and in the control of the transition from vegetative to reproductive growth. Genetic and biochemical experiments reveal that XCT acts with COP1 to regulate ELF3 function in both processes. Our work provides new insights into how signaling components conserved across eukaryotes interact with plant-specific factors to ensure the correct functioning of the circadian system.

## Results

### XCT acts near COP1 and ELF3 to repress the transition to flowering

Mutations that affect the circadian clock often influence photoperiodic regulation of the transition from vegetative to reproductive growth; for example, short-period mutants often flower early in short days but have wild-type flowering phenotypes in long days^40–42^. We therefore investigated whether mutation of *XCT* also affects flowering time regulation. Surprisingly, *xct-2* mutants flower early in long days but do not have a phenotype in short-day conditions (Supplementary Fig. 1). This suggests that this flowering phenotype is not caused by the fast pace of the circadian clock in *xct-2*^43,44^.

**Fig. 1:**
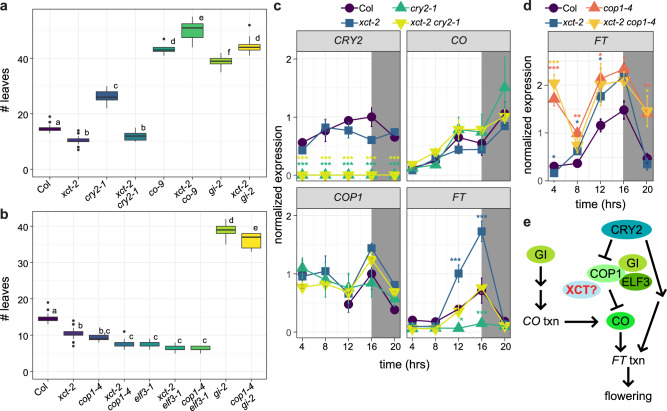
XCT acts near COP1 and ELF3 to regulate flowering time in long days. **a**, **b** Plants were grown in long-day (16 h light:8 h dark) conditions, and the number of leaves produced at the time of bolting was recorded. Different letters denote significant differences between genotypes (*p* < 0.05; one-way ANOVA followed by Tukey’s post hoc test). The lines within the boxes are the medians, and the lower and upper hinges represent the first and third quartiles. *n* = 8–33 plants per genotype; experiment was repeated three times with similar results. **c**, **d** Expression levels of *CRY2*, *CO*, *COP1*, and *FT* were determined by qRT-PCR in the indicated genotypes. Plants were grown in long-day conditions for 10 days and collected at the indicated times relative to lights on. Asterisks indicate expression levels significantly different from Col at the indicated time points (one-way ANOVA; * indicates *p* < 0.05, ** indicates *p* < 0.01, and *** indicates *p* < 0.001). **e** Proposed location of XCT function within the flowering-time pathway. Proteins are indicated with circles; txn indicates regulation of transcription. Arrows indicate promotion, and barred lines indicate inhibition of function. Note that GI promotes *CO* transcription and that, in addition, GI protein stability is regulated by COP1 and ELF3.

We next examined the genetic relationships between *XCT* and a number of genes known to control the transition to flowering in long-day conditions. While plants mutant for the blue light photoreceptor *CRY2* flowered late, *xct-2 cry2-1* mutants flowered with the same number of leaves as *xct-2* (Fig. 1a). This indicates that XCT acts as a repressor of flowering downstream of CRY2 in the floral induction pathway. In contrast, plants mutant for *XCT* and either of the key floral-promoting genes *GI* or *CO* did not flower earlier than *gi-2* or *co-9* single mutants and in fact flowered slightly later (Fig. 1a). The reason for this unexpected genetic interaction is not clear. However, since *XCT* is not required for the late-flowering phenotypes of *co-9* and *gi-2* plants, our data suggest that *XCT* acts upstream of the CO and GI proteins.

Other repressors of flowering previously reported to act downstream of CRY2 and upstream of CO and GI include the E3 ubiquitin ligase COP1 and the scaffold protein ELF3^23^. We therefore examined the genetic interactions between *XCT, COP1*, and *ELF3*. Since null alleles of *COP1* are lethal^45^, we used the reduction-of-function allele *cop1-4* for these studies. We found that in long days, *xct-2*, *cop1-4*, and *elf3-1* single mutants all flowered significantly earlier than wild-type plants (Fig. 1b). *xct-2 cop1-4*, *xct-2 elf3-1*, and *cop1-4 elf3-1* double mutants produced the same number of leaves as the *cop1-4* and *elf3-1* single mutants before transitioning to flowering (Fig. 1b). Although we cannot draw definitive conclusions from this epistasis analysis given that *cop1-4* is not a null allele, the lack of an additive phenotype in these three types of double mutants suggests that *XCT*, *COP1*, and *ELF3* function in the same pathway. Notably, a variety of light signaling and flowering time mutants have been reported to transition to flowering at an even earlier stage of development than *elf3-1* and *cop1-4*^46,47^, suggesting that the lack of additivity in the double mutants is biologically relevant. Together, our genetic analysis suggests that *XCT* acts near *COP1* and *ELF3* to directly or indirectly repress the floral-promoting functions of *GI* and *CO*.

*CO* is a key regulator of flowering that promotes expression of the florigen gene *FT* (Fig. 1e). CO expression is controlled by both transcriptional and post-transcriptional mechanisms; for example, GI promotes transcription of the *CO* gene while COP1 and ELF3 promote degradation of CO protein^19,22,25^. To help determine where in the flowering time pathway *XCT* functions, we used quantitative reverse-transcriptase polymerase chain reaction (qRT-PCR) analysis to examine expression levels of *CO* and *FT*, as well as *CRY2* and *COP1*, in *cry2-1*, *xct-2*, and *xct-2 cry2-1* mutants. We found expression of *CRY2* was not significantly different between wild-type and *xct-2* plants and that *CO* and *COP1* mRNA levels were not significantly different in the four genotypes tested (Fig. 1c). However, and as expected based on previous studies^48^, *FT* levels were significantly reduced in *cry2-1* plants grown in long days (Fig. 1c). Consistent with the early-flowering phenotype of *xct-2* mutants, *FT* levels were significantly elevated in these plants in the late afternoon and at the end of the day (Fig. 1c). *FT* levels were indistinguishable from wild type in *xct-2 cry2-1* mutants, indicating that loss of the negative regulator *XCT* can compensate for mutation of *CRY2* in the regulation of florigen expression.

To further investigate the relationship between COP1 and XCT in control of flowering time, we next examined *FT* mRNA levels in wild-type, *xct-2*, *cop1-4*, and *xct-2 cop1-4* mutants grown in long-day conditions (Fig. 1d). We found that *FT* levels were elevated in *cop1-4* mutants at all time points tested, with significant peaks at both ZT4 and ZT16. Similar to our previous experiment, *FT* levels were most increased in *xct-2* mutants at ZT12 and ZT16 (at the ZT16 time point, *FT* expression in all three mutant genotypes was higher than in wild type with *p* < 0.1), with no upregulation of *FT* at ZT4 or ZT20. These data are consistent with a previous study demonstrating significant upregulation of *FT* in *cop1* mutants grown in long days, likely due to a role for COP1 in a phyA-mediated signaling pathway^19,22,25^. *FT* expression in the *xct-2 cop1-4* double mutants was indistinguishable from that in *cop1-4* mutants, even in the middle and late daytime when *FT* mRNA levels were elevated in both *cop1-4* and *xct-2* single mutants. These gene expression data suggest that XCT and COP1 influence flowering by a similar mechanism during part of the diel cycle but that COP1 has additional, XCT-independent roles in the control of flowering during the late night and early day. Altogether, our genetic and gene expression data suggest that XCT may act near COP1 and ELF3 to modulate CO protein function or stability (Fig. 1e).

### XCT acts near COP1 in the regulation of the circadian clock

**Fig. 2:**
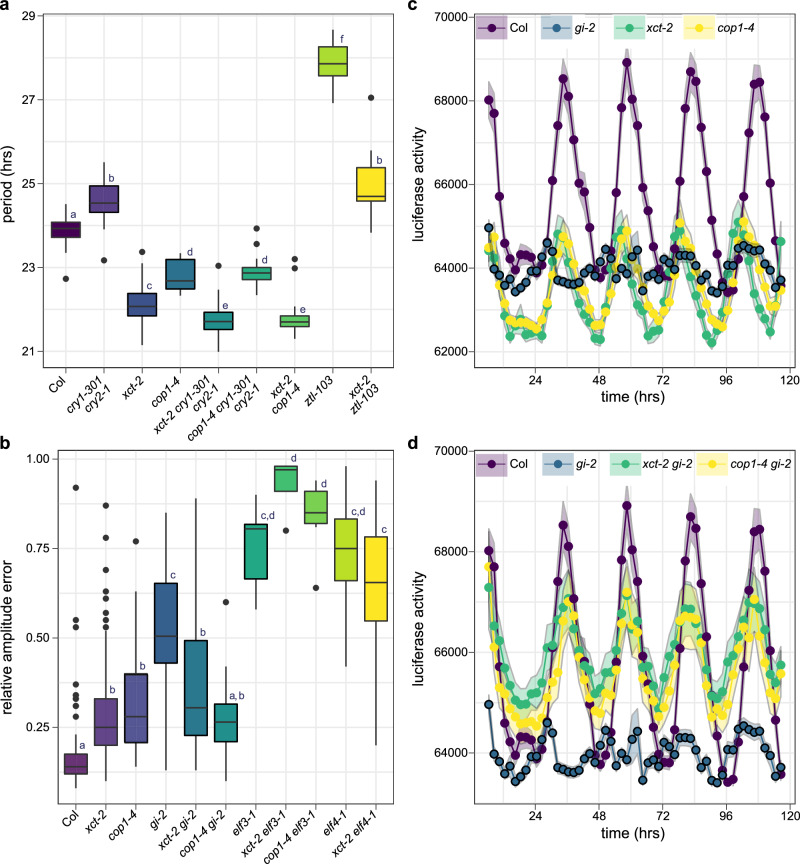
XCT acts near COP1 in the regulation of the circadian clock*.* Luciferase activity was recorded from plants transgenic for *CCR2::LUC+* and maintained in constant light conditions. **a** Median period estimates for all seedlings considered rhythmic (relative amplitude error (RAE) < 0.6). *n* = 12–24 plants. **b** Median RAE for all plants returning a period estimate. *n* = 12–33 plants. **a**, **b** Different letters denote significant differences between genotypes (*p* < 0. 001; one-way ANOVA followed by Tukey’s post hoc test). The lines within the boxes are the medians, and the lower and upper hinges represent the first and third quartiles. **c**, **d** Mean luciferase activities of plants of the indicated genotypes; ribbons indicate means ± SEM. *n* = 19–25 plants per genotype. The experiment was repeated three times with similar results.

We next examined the genetic relationships between *XCT* and other genes that act within or near the circadian oscillator. To assess circadian clock function, we introduced a transgene carrying the *COLD, CIRCADIAN RHYTHM, AND RNA BINDING 2* promoter driving expression of firefly luciferase (*CCR2::LUC*+) into various mutant backgrounds by crossing. After entrainment, plants were moved to constant environmental conditions and luciferase activity was monitored for several days, allowing us to assess circadian rhythms in reporter gene expression. First, we examined the genetic interactions between *XCT* and *COP1*. As expected^28,33^, *xct-2* and *cop1-4* mutants had short-period phenotypes in this assay. Intriguingly, the free-running period of *xct-2 cop1-4* double mutants was not significantly different than that of *xct-2* single mutants (Fig. 2a). This suggests that XCT and COP1 act together to control circadian period. We next examined the phenotypes of plants mutant for *XCT* or *COP1* and blue light photoreceptors known to affect clock pace. Consistent with previous reports^49,50^, we found that plants double-mutant for *CRY1* and *CRY2* or mutant for the circadian clock-specific blue light photoreceptor *ZEITLUPE* (*ZTL*) exhibited long-period phenotypes (Fig. 2a). We found that the period of *xct-2 ztl-103* mutants was intermediate between each of the parental single mutants (Fig. 2a), suggesting that XCT and ZTL affect clock pace via parallel pathways. In contrast, the phenotypes of the *xct-2 cry1-301 cry2-1* and *cop1-4 cry1-301 cry2-1* triple mutants were not significantly different from the *xct-2* and *cop1-4* single mutants, respectively. Overall, these genetic data suggest that both *COP1* and *XCT* act downstream of *CRY1* and *CRY2* to control clock pace.

In addition to affecting the pace of the circadian clock, mutation of circadian genes often affects the rhythmic robustness of the circadian oscillator. This can be quantified by assessing the relative amplitude error (RAE) associated with luciferase activity rhythms, with an RAE of 0 indicating highly rhythmic expression patterns and an RAE of 1 indicating no statistically significant rhythmic element is present. For example, null mutations in either *ELF3* or *ELF4*, another component of the Evening Complex (EC), result in very high RAE values (Fig. 2b), indicating these plants are nearly arrhythmic^51,52^. We therefore examined genetic interactions between circadian clock gene mutants and *xct-2* and *cop1-4* on rhythmic robustness. Mutation of *XCT* or *COP1* caused modest but statistically significant increases in RAE values. The *xct-2 elf3-1* and *xct-2 elf4-1* plants had similarly high RAE values as the *elf3-1* and *elf4-1* single mutants (Fig. 2b). This non-additivity is consistent with XCT acting in a similar position within the oscillator as these EC components.

GI is another clock component reported to interact with both COP1 and ELF3^23^. We found that a null mutation in *GI* caused a significant reduction in rhythmicity and decreased luminescence levels relative to wild type (Fig. 2b, c), consistent with previous reports^53,54^. To our surprise, we found that combining this *gi-2* mutation with either *xct-2* or *cop1-4* significantly increased the robustness of *CCR2::LUC+* rhythms and overall luminescence when compared to the *gi-2* single mutant (Fig. 2b, d, Supplementary Fig. 2a–c). This demonstrates that *XCT* and *COP1* are epistatic to *GI* in control of rhythmic robustness. *xct-2* and *cop1-4* were also largely epistatic to *gi-2* in their effects on free-running circadian period (Supplementary Fig. 2a). The improved rhythmicity in *xct-2 gi-2* and *cop1-4 gi-2* relative to the null allele *gi-2*^53^ suggests that *XCT* and *COP1* may act antagonistically to *GI* in the regulation of a shared circadian regulatory component or process. Overall, these genetic data suggest that *XCT* and *COP1* act in a similar manner to affect the circadian oscillator.

### XCT interacts with COP1 and negatively regulates ELF3 protein abundance

**Fig. 3:**
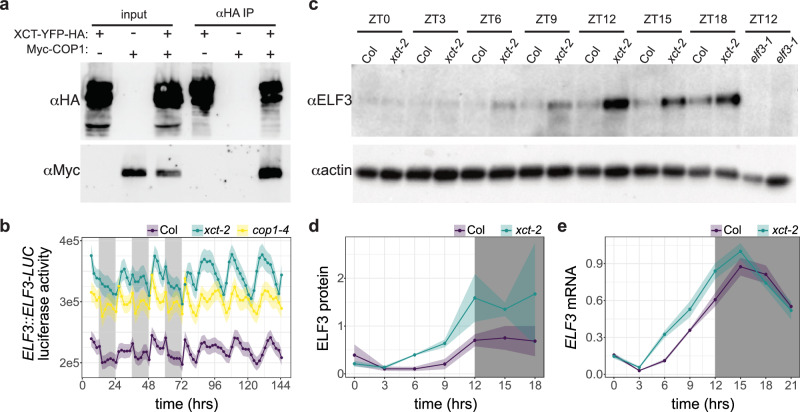
XCT physically interacts with COP1 and regulates ELF3 protein levels. **a** XCT and COP1 co-immunoprecipitate. Protein extracts were made from *N. benthamiana* leaves agroinfiltrated with the indicated expression vectors. Expressed XCT-YFP-HA and Myc-COP1 were detected with anti-HA and anti-Myc antibodies (input). Co-purification of Myc-COP1 with XCT-YFP-HA (αHA IP) was detected by immunoblotting. **b**–**e** XCT and COP1 negatively regulate ELF3 accumulation in vivo. **b** Luciferase activity from Col, *xct-2*, and *cop1-4* plants expressing a translational fusion between ELF3 and LUC under the control of the native *ELF3* promoter in light/dark cycles and constant light. **c** Abundance of native ELF3 protein in nuclear extracts of wild-type and *xct-2* plants in light/dark cycles as detected with an anti-ELF3 antibody. Nuclear extracts of *elf3-1* plants were included as a negative control, and actin was detected with an anti-actin antibody as a loading control. **d** Quantification of anti-ELF3 immunoblots (ribbon indicates ± SEM; *n* = 2). The amplitude of a cosinar model^57^ fit to rhythms in ELF3 protein levels is 2.2× higher in *xct-2* than in Col (*p* < 0.05). **e** Expression levels of *ELF3* mRNA were determined by qRT-PCR. Plants were grown in 12 h light/12 h dark conditions for 10 days and collected at the indicated times relative to lights on. No significant difference in amplitude (*p* > 0.05) was detected between the two genotypes. **c**–**e** Plants were grown in 12 h light:12 h dark conditions for 10 days and collected at the indicated time points relative to lights on. **b**, **d**, **e** Gray boxes indicate times plants were grown in darkness. Experiments were repeated 2–3 times with similar results.

Since *xct* and *cop1* share similar phenotypes and genetic interactions, we next investigated whether COP1 and XCT proteins physically interact. We transiently expressed epitope-tagged COP1 and XCT in *Nicotiana benthamiana* leaves and then carried out co-immunoprecipitation reactions followed by immunoblotting. We found that COP1 co-purified with XCT (Fig. 3a), suggesting that these two proteins might work together in vivo to regulate pathways such as flowering time regulation and the circadian oscillator.

COP1 has previously been shown to regulate flowering time and circadian clock function via a physical interaction with ELF3, which leads to a COP1-dependent decrease in ELF3 protein abundance^23,55^. Given the physical interaction between COP1 and XCT, we speculated that XCT might also modulate ELF3 protein levels. To test this, we first introduced a transgene with the Arabidopsis *ELF3* promoter controlling transcription of a translational fusion between ELF3 and luciferase (*ELF3::ELF3-LUC*)^56^ from wild type into *cop1-4* and *xct-2* plants by crossing. In light/dark cycles, wild-type plants displayed a complex pattern of luciferase activity that in constant light conditions resolved into a circadian pattern with peak expression during the subjective night (Fig. 3b). *cop1-4* mutants displayed a similar overall pattern of luciferase activity, but with substantially higher luminescence than wild type. These results are consistent with previous reports that COP1 mediates proteasome-dependent degradation of ELF3^23,55^. *xct-2* mutants expressed significantly higher levels of *ELF3-LUC* than wild type and slightly more than *cop1-4*. This suggests that, like COP1, XCT may negatively regulate ELF3 protein levels.

To test the effects of the *xct-2* mutation on the abundance of native ELF3 protein, we next performed immunoblotting experiments with anti-ELF3 antibody on nuclear extracts prepared from plants grown in 12 h light/12 h dark cycles. We found higher levels of ELF3 protein in *xct-2* than in Col, particularly in the late afternoon and the first part of the night (Fig. 3c). Cosinor analysis of ELF3 protein abundance with CircaCompare^57^ revealed a greater than two-fold higher amplitude of ELF3 protein level oscillations in *xct-2* than in control plants (Fig. 3d; Supplementary Data 1). We next investigated whether this difference might be due to increased *ELF3* transcript levels in *xct-2* mutants. qRT-PCR analysis of *ELF3* mRNA levels in plants maintained in light/dark cycles revealed a phase advance in the accumulation of *ELF3* transcript in *xct-2* mutants relative to wild type (Fig. 3e), presumably due to the short-period phenotype of these plants^33^. However, we did not detect a significant difference in the amplitude of *ELF3* mRNA level oscillations in *xct-2* and Col (Supplementary Data 1). Together, these results indicate that XCT negatively regulates ELF3 protein abundance, most likely by collaborating with COP1 to promote ELF3 degradation.

### XCT promotes ELF3 activity

**Fig. 4:**
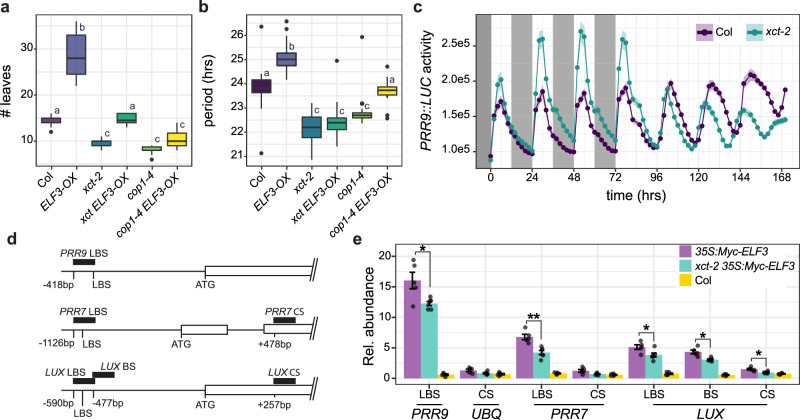
XCT promotes ELF3 activity. **a**, **b** *XCT* and *COP1* are required for *ELF3-OX* phenotypes. **a** Plants were grown in long-day (16 h light:8 h dark) conditions, and the number of leaves produced at the time of bolting was recorded. *n* = 9–19 plants. **b** Luciferase activity was recorded from plants transgenic for *CCR2::LUC+* and maintained in constant light conditions. Median period estimates for all seedlings considered rhythmic (relative amplitude error (RAE) < 0.6). *n* = 21–40 plants. **a**, **b** The lines within the boxes are the medians, and the lower and upper hinges represent the first and third quartiles. Different letters denote significant differences between genotypes (*p* < 0.001; one-way ANOVA followed by Tukey’s post hoc test). **c** Luciferase activity was recorded from plants transgenic for *PRR9::LUC2*, first growing in light/dark cycles, then in constant light conditions. Mean luciferase activities are plotted; ribbons indicate means ± SEM. Gray boxes indicate times plants were in darkness. *n* = 34–35 plants. **d**, **e** XCT promotes ELF3 association with chromatin. **d** Schematic of *PRR9*, *PRR7*, and *LUX* genomic regions; black boxes indicate regions amplified by qRT-PCR. LUX BINDING SITE (LBS), non-canonical LUX BINDING SITE (BS), CODING SEQUENCE (CS), and translational start codons (ATG) are indicated. Numbers indicate nucleotide positions of each amplicon relative to the translational start codons. **e** Relative mean abundance of immunoprecipitated DNA (as percent of input DNA × 10e^−3^) corresponding to the indicated regions of the *LUX*, *PRR7*, and *PRR9* genes is plotted. The promoter of the *UBQ10* was amplified as a negative control ± SEM, *n* = 5–7. Values of individual replicates are plotted as points. (* indicates *p* < 0.05, ** indicates *p* < 0.01, Welch’s one-way ANOVA). Experiments were repeated 2–3 times with similar results.

While COP1 mediates the proteasomal degradation of ELF3 protein, it is (somewhat counter-intuitively) also required for ELF3 protein function^23^. Given that *cop1-4* and *xct-2* demonstrate similar genetic interactions in the control of flowering time and the circadian clock (Figs. 1 and 2), we next investigated whether XCT might also be required for ELF3 activity. As expected given previous reports^23,58^, we found that plants overexpressing *ELF3* flowered later than wild-type in long days, but that *cop1-4 ELF3-OX* plants flowered as early as *cop1-4* mutants (Fig. 4a). *xct-2 ELF3-OX* plants flowered with a number of leaves indistinguishable from wild type (Fig. 4a). These results indicate that COP1 and, to a lesser extent, XCT are required for the effects of ELF3 on the regulation of the transition from vegetative to reproductive growth.

We next examined genetic interactions between *ELF3-OX*, *COP1*, and *XCT* on the regulation of circadian clock pace. We found that the long-period phenotype of *ELF3-OX* plants^59^ was partially suppressed by the *cop1-4* mutation, with *cop1-4 ELF3-OX* plants having a period phenotype indistinguishable from wild type and intermediate between *cop1-4* and *ELF3-OX* mutants (Fig. 4b). The suppressive effects of *xct-2* were stronger, with *xct-2 ELF3-OX* plants having a short-period phenotype indistinguishable from *xct-2* single mutants (Fig. 4b). This epistasis indicates that XCT and to a lesser extent COP1 are required for ELF3 control of clock pace.

ELF3 and other EC components directly negatively regulate the expression of key clock genes such as *PRR7*, *PRR9*, and *LUX*^12,13,60^. To investigate whether XCT might affect EC target gene expression in an environmentally-responsive manner, we introduced a *PRR9::LUC2* reporter gene into *xct-2* plants by crossing with wild type. This transgene confers early day-phased rhythms in luminescence in plants, consistent with the accumulation patterns of *PRR9* mRNA^61^. In 12 h light: 12 h dark cycles, peak levels of *PRR9::LUC2* activity were much higher in *xct-2* than in control plants (Fig. 4c). However, after 24 h in constant light conditions, peak levels of luminescence were very similar between the two types of seedlings. qRT-PCR experiments also revealed higher peak levels of *PRR9* expression in *xct-2* compared to controls in plant grown in light/dark conditions (Supplementary Fig. 3). These data are consistent with our previous finding that *PRR7* and *PRR9* levels are elevated in *xct-2* plants maintained in short-day conditions but not in constant light^33^ and suggest the ability of the EC to repress gene expression may be reduced in the absence of XCT.

To investigate possible ways in which the ability of ELF3 to control gene expression might be altered in *xct* mutants, we carried out chromatin immunoprecipitation (ChIP) experiments. A transgene encoding epitope-tagged ELF3 expressed under the control of the 35S promoter was introduced into *xct-2* by crossing with wild type. Plants were grown in 12 h light: 12 h dark cycles, and samples were harvested shortly before dawn. Immunoprecipitations were carried out using anti-Myc antibodies, with non-transgenic Col seedlings serving as a negative control. The abundance of genomic DNA in regions of the *PRR7*, *PRR9*, and *LUX* promoters known to serve as LUX binding sites (LBS) as well as regions within the coding sequences of these genes not expected to associate with the EC (Fig. 4d) was assessed by qRT-PCR. We found that regions containing known EC binding sites were considerably more abundant in both wild-type and *xct-2* plants expressing Myc-tagged ELF3 than in non-transgenic controls (Fig. 4e). However, in *xct-2* plants, the enrichment of *PRR9* and *PRR7* promoter sequences in ELF3 chromatin immunoprecipitations was significantly lower than in plants expressing functional *XCT* (Fig. 4e, Supplementary Data 2). Given that ELF3 protein levels are actually elevated in *xct-2* relative to wild type (Fig. 3c, d), these data indicate that XCT promotes ELF3 association with chromatin despite its negative effects on ELF3 protein levels.

## Discussion

Although the pathways regulating flowering time and the circadian clock are well-studied, the underlying molecular mechanisms are still being elucidated. Here, we shed light on the role of XCT, a nuclear protein conserved across eukaryotes, in these processes. Our genetic analyses reveal that XCT acts downstream of CRY2 and upstream of GI and CO in the control of flowering time. The normal levels of *CO* mRNA in *xct-2* mutants combined with elevated levels of *FT* transcript (Fig. 1) suggest that XCT controls CO protein stability or function, activities previously ascribed to COP1^23^. Consistent with XCT acting with COP1, our genetic analysis indicates XCT acts downstream of cryptochromes and near COP1 in control of circadian clock pace (Fig. 2). Since COP1 has previously been shown to act with ELF3 to control flowering time and the circadian oscillator^23^, we investigated possible interactions between XCT and COP1. We found that XCT physically interacts with COP1, and like COP1, negatively regulates ELF3 protein levels (Fig. 3). Counterintuitively, but also like COP1, XCT promotes ELF3 activity, enhancing its ability to bind chromatin (Fig. 4). Together, these data suggest that XCT works with COP1 to promote ELF3 function, ensuring normal circadian activity and control of flowering time.

Null mutations in *ELF3* cause circadian arrhythmicity, very different from the short-period phenotype seen in *xct* mutants^33,51^. However, there are intriguing similarities between plants mutant for *XCT* and plants with reduced *ELF3* activity: as seen in *xct-2*, the hypomorphic allele *elf3-12* causes a short-period phenotype and modestly increased hypocotyl length in constant light^33,62^. In addition, the circadian clock in both *xct-2* and *elf3-12* mutants is hypersensitive to light cues given between the late subjective night and the early subjective day^33,62^. These results support our conclusion that XCT normally promotes ELF3 activity within the circadian system.

Recent publications also support our findings linking XCT and COP1. It has long been recognized that COP1 regulates gene expression via the regulated degradation of transcription factors and other signaling proteins^63^. However, two recent papers have shown that COP1 is also directly involved in the regulation of RNA splicing^64,65^. COP1 modulates alternative splicing in a light-dependent manner, with mutations in several conserved spliceosomal proteins suppressing *cop1* growth phenotypes^65^. Intriguingly, a recent cryoEM structure of the human spliceosome shows that FAM50a, the human homolog of XCT, physically interacts with several of these COP1 suppressor proteins^66^. In support of this structural data, two of these suppressor proteins have been shown to co-purify with FAM50a^34^. This raises the interesting possibility that these spliceosomal proteins may also be important for XCT activity.

XCT is highly conserved across eukaryotes, with homologs found in most eukaryotic genomes^33,38^. In fact, the Arabidopsis protein can complement the growth phenotype of *Schizosaccharomyces pombe* deficient for *Xap5*, the fission yeast ortholog of XCT^37^. The C-terminal half of XCT is the most conserved region, with ~65% amino acid identity observed between these portions of the Arabidopsis, human, and *Chlamydomonas reinhardtii* proteins (Supplementary Fig. 4a). In contrast, the N-terminal regions of XCT orthologs show considerably less sequence similarity across diverged taxa. However, analysis of the primary protein sequences of the Arabidopsis, human, *Chlamydomonas reinhardtii*, and fission yeast XCT homologs suggests that their N-termini all have a high degree of intrinsic disorder (Supplementary Fig. 4b^67^). Many intrinsically disordered proteins play important roles in processes including RNA processing and the regulation of transcription^68–70^. The combination of an unstructured region at the N-terminus and a highly conserved amino acid domain at the C-terminus is likely important for XCT function in diverse organisms.

We and others have reported roles for XCT and its homologs in both RNA splicing and the regulation of transcription^34–38^. This raises the obvious question as to whether XCT regulates ELF3 activity via RNA processing. For several reasons, we believe that XCT affects clock function independently of its role in RNA splicing. First, we do not observe differential splicing of *COP1*, *ELF3*, or known targets of ELF3 in *xct* mutants^36^. Second, the physical interaction between COP1 and XCT, plus the increased ELF3 protein levels in *xct-2* in the absence of altered *ELF3* transcript levels, suggests that XCT directly regulates ELF3 protein. Third, we previously found that circadian clock function but not RNA splicing is rescued in plant mutants for both *XCT* and either one of the two Arabidopsis *PRECURSOR RNA PROCESSING 19*(*PRP19*) paralogs, demonstrating that the XCT splicing and circadian phenotypes are genetically separable^36^. Finally, the *Chlamydomonas reinhardtii* homolog of XCT can bind directly to gene promoters and to RNA Pol II^38^, demonstrating that members of this gene family may play a direct role in transcriptional regulation. Taking all these findings together, we conclude that XCT acts with COP1 to regulate ELF3 activity independently of its role in RNA processing.

While transcription and RNA processing are inextricably coupled to each other^71,72^, separable roles for proteins in these two processes are not uncommon. For example, although the PRP19 complex is best known for its role in the spliceosome, several components of this complex control transcription through binding to the promoters of genes and to transcription factors, chromatin-modifying enzymes, or RNA polymerase II^73–78^. Intriguingly, Arabidopsis PRP19A itself has recently been shown to modulate gene expression by competing with the transcription factor ELONGATED HYPOCOTYL 5 (HY5) for binding to the regulatory regions of shared target genes in a CRY2-regulated manner^79^. Separable roles for XCT in transcriptional regulation and RNA processing are therefore far from unprecedented. It will be very interesting to determine the extent of conservation of these distinct roles for XCT across eukaryotes and to understand how these nuclear activities are coordinated with each other.

## Methods

### Plant materials

All plants used are in the Columbia (Col-0) background. The following genotypes have been previously described: *co-9*^80^; *cop1-4*^45^; *cry1-301*^81^; *cry2-1*^24^; *elf3-1*^59^; *elf4-1*^52^ (this mutation was originally isolated in the Ws accession but was introgressed into Col-0 by four generations of backcrossing); *gi-2*^82,83^; *xct-2*^33^; and *ztl-103*^84^. The *CCR2::LUC*+, *PRR9::LUC2*, and *ELF3::ELF3-LUC* reporters have been previously described^41,56,85^. Myc-tagged, *ELF3*-overexpressing plants were generated as follows. The *ELF3* cDNA was amplified (see primer sequences in Supplementary Data 3) and cloned into the pEarleyGate 203 transformation vector^86^ using LR Clonase reaction mix (Invitrogen). Transformation of Col-0 plants was achieved using the floral dip method^87^ and transgenic T1 seedlings selected using the herbicide Basta. Late-flowering lines were selected, and *ELF3* overexpression was verified by immunoblotting. Double-mutant genotypes were generated by crossing. All transgenes were introduced into mutant backgrounds by crossing with the corresponding wild-type controls.

### Growth conditions

Seeds were surface-sterilized with chlorine gas and stratified in the dark for 2–4 days at 4 °C. For luciferase imaging, seeds were plated on 1× Murashige and Skoog media, 0.7% agar, and 3% sucrose. Seedlings were entrained in light-dark cycles (12 h light, 12 h dark) with 50–60 μmol m^−2^ s^−1^ cool white fluorescent light at 22 °C for 6 days. For chromatin immunoprecipitation experiments, seeds were plated on 1× Murashige and Skoog media, 0.7% agar, 1% sucrose and grown in light–dark cycles (12 h light, 12 h dark) with 50–60 μmol m^−2^ s^−1^ cool white fluorescent light at 22 °C for 12 days and then at 17 °C for 2 additional days. Plants were harvested at ZT23.5.

For flowering time, immunoblotting, and quantitative PCR experiments, seeds were sown directly in soil, and plants were grown under light–dark cycles of the specified photoperiod with 150–200 μmol m^−2^ s^−1^ cool white fluorescent light at 22 °C. For immunoblotting and quantitative PCR, plants were collected at the indicated times and immediately frozen in liquid nitrogen. For flowering assays, rosette leaves were counted when inflorescence stems were 1 cm long.

### Quantitative PCR

Sample preparation and qRT-PCR were performed as previously described^88^ using a BioRad CFX96 thermocycler (Bio-Rad Laboratories). Relative expression and SEM values were obtained from the BioRad CFX96 software package. Primer sequences are in Supplementary Data 3.

### Chromatin immunoprecipitation

ChIP assays were performed primarily as previously described^89^. Briefly, Arabidopsis seedlings were harvested and fixed with 1% formaldehyde under vacuum for 10 min. For each immunoprecipitation, nuclei were isolated from 2.5 g of seedling tissue, lysed, and then chromatin was sheared into fragments with an average length of 100–500 bp using a Covaris E220 sonicator (Covaris). Chromatin was incubated with 4.5 μg of mouse monoclonal anti-Myc antibody (9E 10; Developmental Studies Hybridoma Bank) overnight at 4 °C. Ten micrograms of rabbit anti-mouse secondary antibody (#12-488, Millipore Sigma) and protein A Dynabeads (#10001, ThermoFisher) were then added. Input and purified, immunoprecipitated DNA were quantified by real-time PCR. The sequences of the primers used are listed in Supplementary Data 3.

### Co-immunoprecipitation and immunoblotting

For the co-immunoprecipitation experiments, XCT-YFP-HA and Myc-COP1 were transiently expressed in *Nicotiana benthamiana*. The *XCT-YFP-HA* plasmid has been previously described^33^; the *Myc-COP1* plasmid was generated by amplifying the *COP1* cDNA (see primer sequences in Supplementary Data 3) and cloning it into the pEarleyGate 203 transformation vector^86^ using LR clonase reaction mix (Invitrogen). Leaf infiltrations were carried out largely as described^90^ except that *Agrobacterium tumefaciens* GV3101 strain was diluted to an OD = 0.1 in 10 mM MgCl_2_ for infiltration and tissue was harvested 3 days after infiltration. Extraction of nuclear proteins from *Nicotiana benthamiana* and Arabidopsis and anti-HA immunoprecipitations were carried out as previously described^36^. Nuclear lysates or immunoprecipitated samples were analyzed by immunoblotting. The primary antibodies used were: rabbit anti-ELF3^14^; mouse anti-Myc (#ZMS1032, Millipore Sigma); mouse anti-HA conjugated to HRP (#12013819001, Millipore Sigma); and mouse anti-actin (#A0480, Millipore Sigma). The secondary antibodies used were: goat anti-mouse HRP (#31430, ThermoFisher Scientific) and goat anti-rabbit HRP (#31460, ThermoFisher Scientific). HRP activity was determined using SuperSignal West Pico chemiluminescent substrate (#34580, ThermoFisher Scientific) and protein abundance quantified using Image Lab software (Bio-Rad Laboratories).

### Circadian period analysis

After entrainment in light-dark cycles, seedlings were sprayed with 3 mM D-luciferin (Gold Biotechnology) and moved to the specified light conditions using red and blue LEDs (35 μmol m^−2^ s^−1^ each color; XtremeLUX, Santa Clara, CA) to provide illumination. Plants were imaged for 5–6 days using a cooled CCD camera (DU434-BV, Andor Technology; or ORCA-R2, Hamamatsu Photonics). Bioluminescence was quantified using MetaMorph software (Molecular Devices) and circadian rhythmicity assessed using Biological Rhythm Analysis Software System (BRASS)^91^.

### Disorder analysis

Protein sequences of the *Arabidopsis thaliana*, *Homo sapiens*, *Chlamydomonas reinhardtii*, and *Schizosaccharomyces pombe* XCT homologs were obtained from Uniprot (accession numbers Q8H110, Q14320, A0A159YK42, and Q7LKZ5). Protein alignments were generated using Clustal Omega^92^ and viewed and edited using Jalview 2^93^. Predictions of disordered domains were obtained from the Database of Disordered Protein Predictions (d2p2)^67^. d2p2 uses nine predictor algorithms (VLXT, VSL2b, PrDOS, PV2, IUPred-S, IUPred-L, Espritz-N, Espritz-X, and Espritz-D^94–99^) and requires that 75% of the programs agree before defining a region as disordered.

## Supplementary information

Supplementary Information

Supp_Data_1

Supp_Data_2

Supp_Data_3

Transparent Peer Review file

## Data Availability

All data generated during the study are either included in the supplementary files or are available from the corresponding author upon reasonable request.
