# Supplementary material for: Conserved eukaryotic factors XCT and COP1 work together to control circadian clock function and reproductive timing in plants
Source: NPJ Biol Timing Sleep. 2026 Jan 9;3:2. doi: 10.1038/s44323-025-00057-x (PMC12789028; doi:10.1038/s44323-025-00057-x)
Supplement: Supplementary file 1 — Supplementary Information [file 44323_2025_57_MOESM1_ESM.pdf]

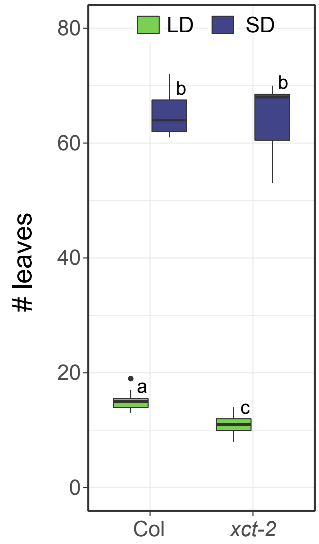

Supplementary Figure 1. **XCT acts as a repressor of flowering in long days**. Plants were grown in either long-day (LD; 16 hour light:8 hour dark) or short-day (SD; 8 hour light:16 hour dark) conditions. The number of leaves produced at the time of bolting was recorded. Different letters denote significant differences between genotypes ($p < 0.05$), determined by one-way ANOVA followed by Tukey's post hoc test. The lines within the boxes are the medians, and the lower and upper hinges represent the first and third quartiles. n = 13 - 19 plants per condition.

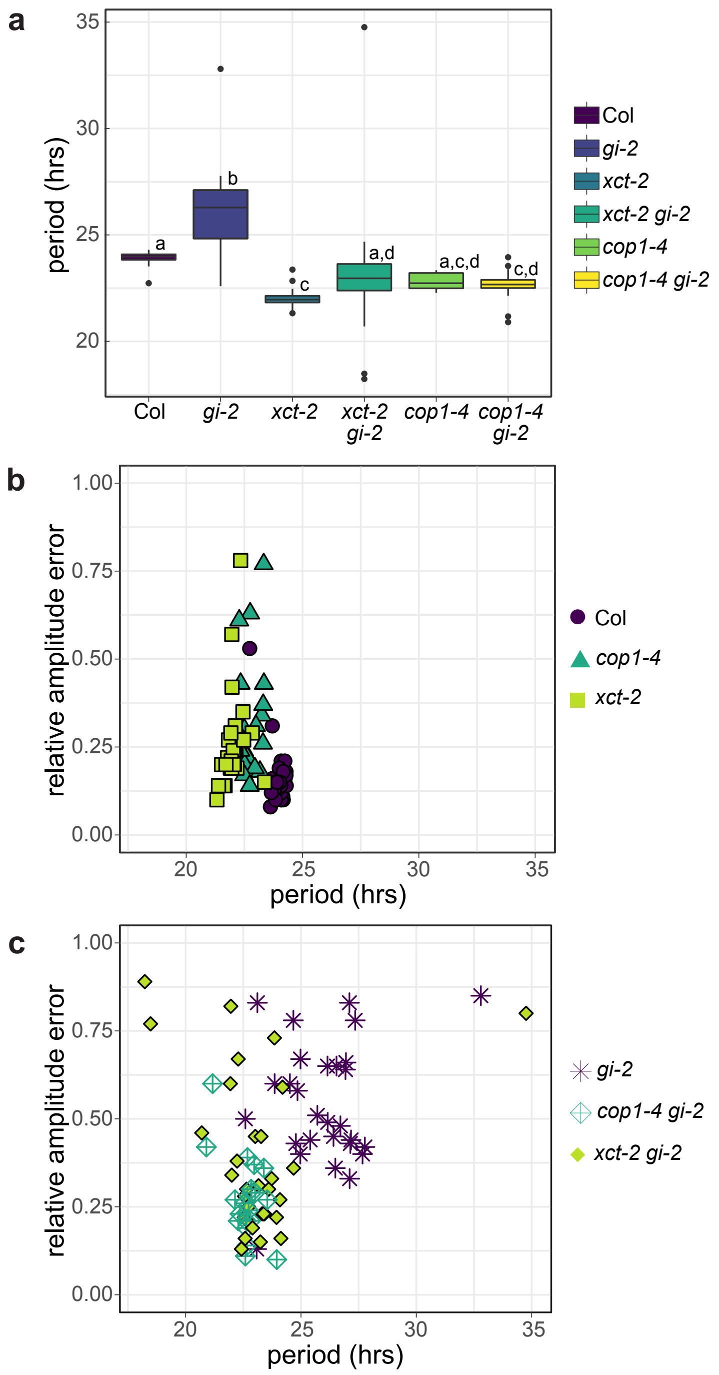

Supplementary Figure 2. *XCT* and *GI* have antagonistic effects on circadian robustness. Luciferase activity was recorded from plants transgenic for *CCR2::LUC*+ and maintained in constant light conditions. **a**) Median period estimates for all seedlings considered rhythmic (relative amplitude error (RAE) < 0.6). Different letters denote significant differences between genotypes (p < 0. 001; one-way ANOVA followed by Tukey's post hoc test). The lines within the boxes are the medians, and the lower and upper hinges represent the first and third quartiles. **b**) and **c**) Period and relative amplitude error are plotted for all plants that returned period estimates. n = 19 – 36 plants per genotype. Experiment was repeated three times with similar results.

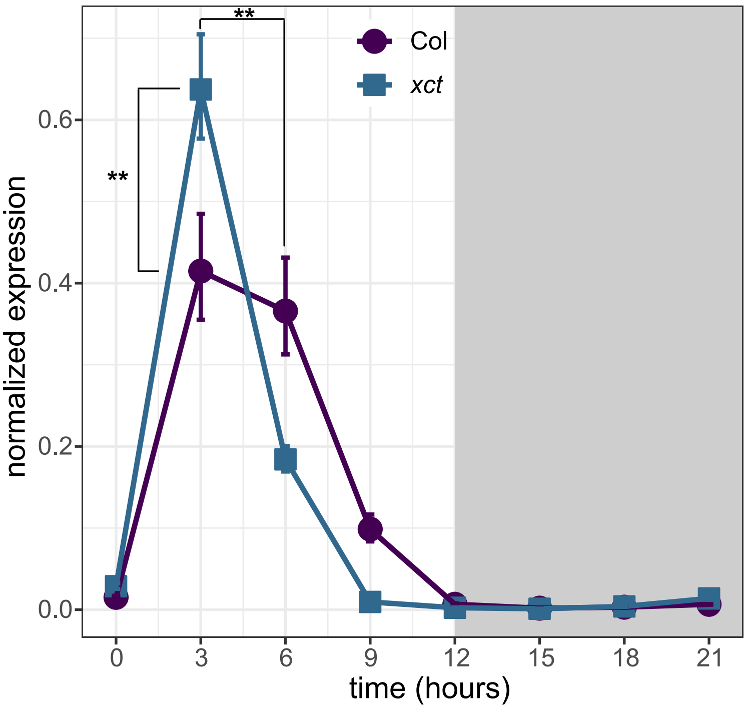

Supplementary Figure 3. *PRR9* mRNA levels are elevated in *xct* mutants. Expression levels of *PRR9* were determined by qRT-PCR. Plants were grown in 12 hour light: 12 hour dark conditions for 10 days and samples were collected at the indicated times relative to lights on. Mean values of two independent replicates are plotted SEM. Asterisks indicate expression levels significantly different between Col and *xct-2* at the indicated time points (one-way ANOVA followed by Tukey's post-hoc test, ** indicates p < 0.01). Grey box indicates time plants were in darkness.

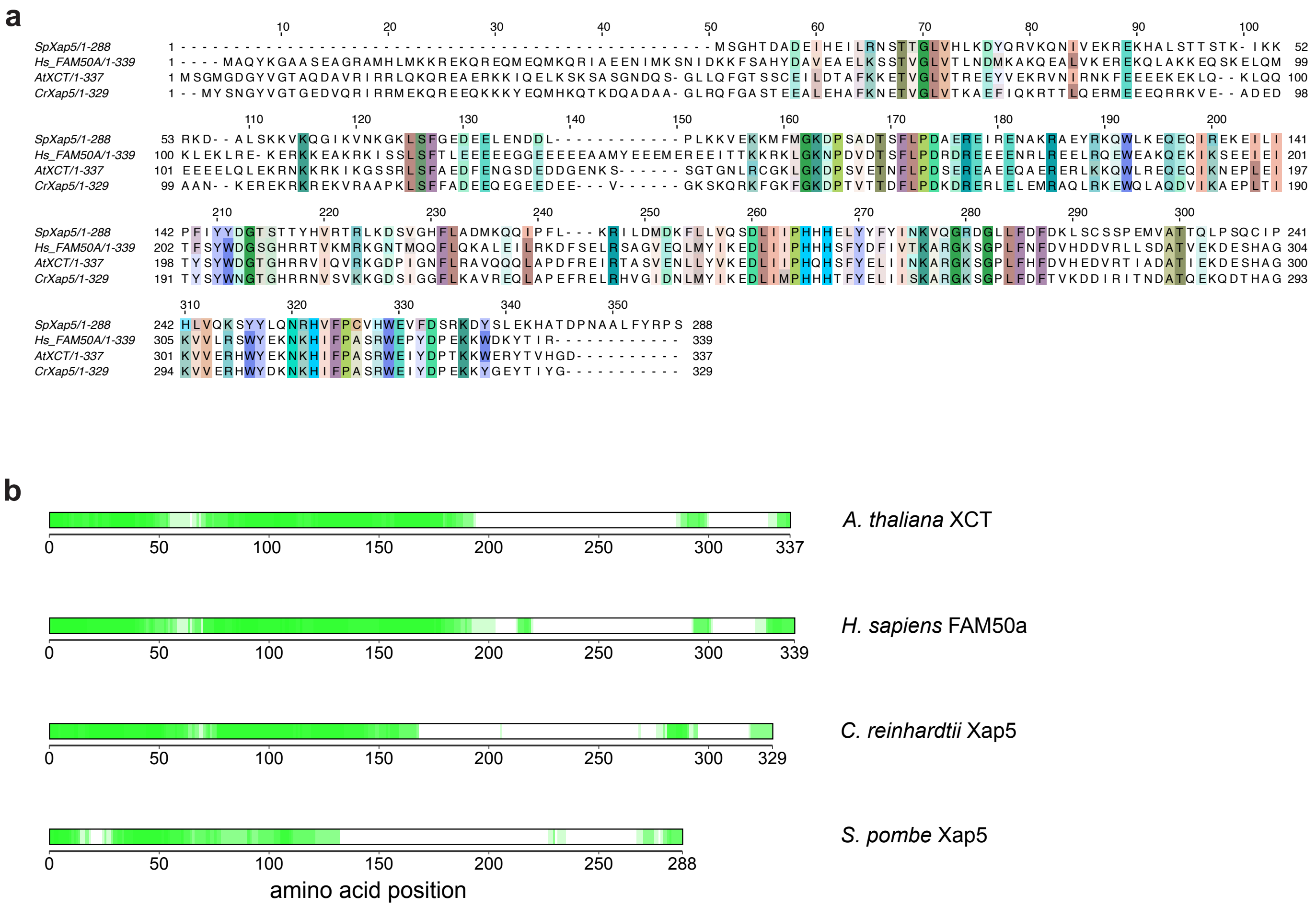

Supplementary Figure 4. Conservation of sequence and predicted disorder regions in XCT homologs. a) Alignment of XCT-like proteins from *Schizosaccharomyces pombe*, *Homo sapiens*, *Arabidopsis thaliana*, and *Chlamydomonas reinhardtii*. Amino acids are colored to illustrate degree of conservation. **b**) Prediction of disordered regions in XCT homologs by the d2p2 algorithm. Green blocks indicate regions predicted to be disordered by multiple algorithms. Note that although the N-termini of these proteins have limited sequence conservation, they are all predicted to be disordered.

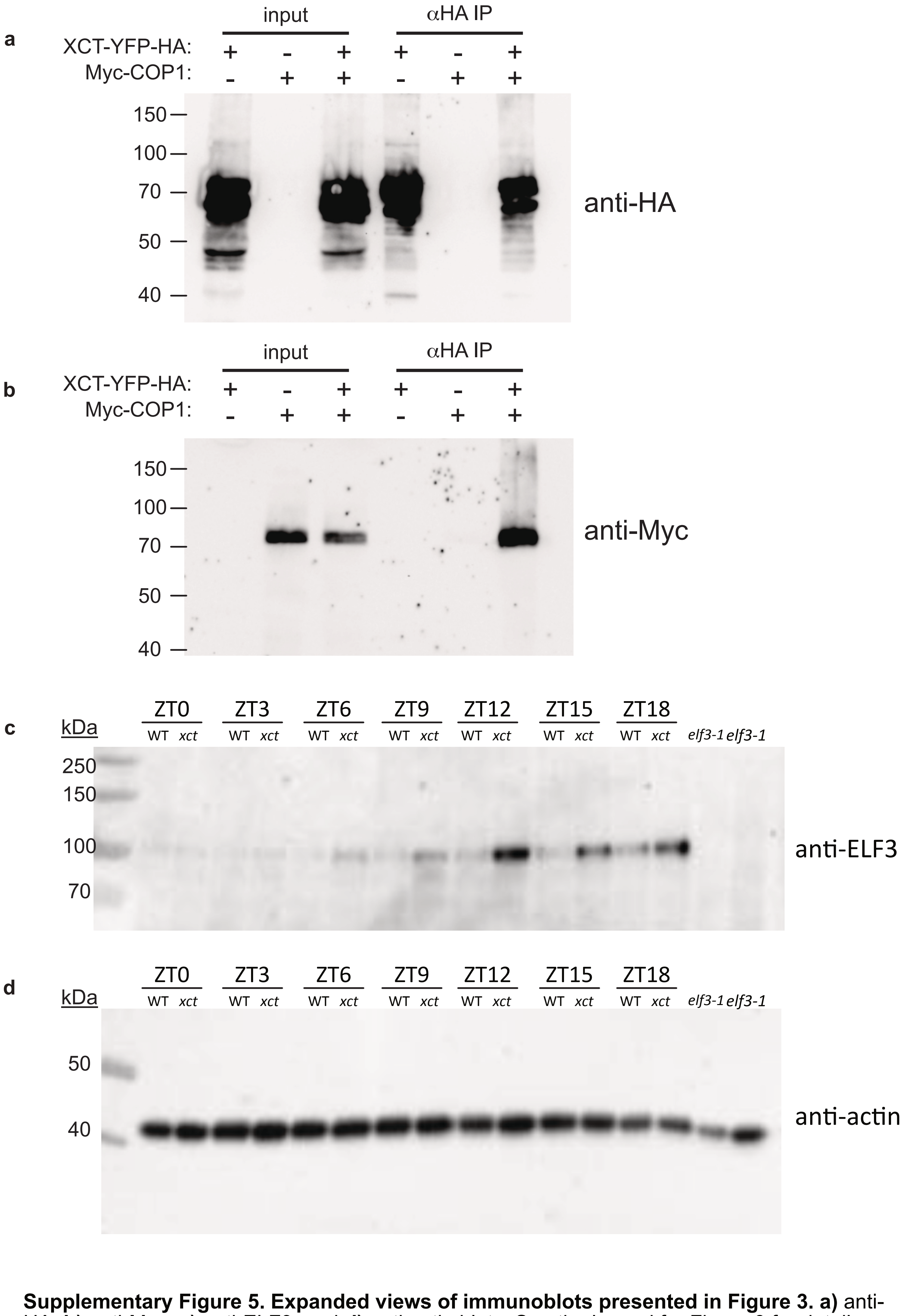

Supplementary Figure 5. Expanded views of immunoblots presented in Figure 3. a) anti-HA, **b)** anti-Myc, **c)** anti-ELF3, and **d)** anti-actin blots. See the legend for Figure 3 for details.
