# Supplementary material for: Conserved eukaryotic factors XCT and COP1 work together to control circadian clock function and reproductive timing in plants
Source: NPJ Biol Timing Sleep. 2026 Jan 9;3:2. doi: 10.1038/s44323-025-00057-x (PMC12789028; doi:10.1038/s44323-025-00057-x)
Supplement: Supplementary file 5 — Transparent Peer Review file [file 44323_2025_57_MOESM5_ESM.docx]

**Supplementary Data 1. Statistical analysis of rhythmic amplitude of ELF3 mRNA and protein.** Rhythmic parameters for ELF3 mRNA and protein extracted from plants grown in 12 hour light:12 hour dark cycles (data plotted in Fig. 3). Analysis was performed using CircaCompare ^57^.

**Supplementary Data 2. Values of independent replicates for the ChIP experiment shown in Fig. 4e.**

**Supplementary Data 3. Sequences of oligonucleotides used in this study.**
